# Predicting osteoporotic fractures post-vertebroplasty: a machine learning approach with a web-based calculator

**DOI:** 10.1186/s12893-024-02427-x

**Published:** 2024-05-09

**Authors:** Sanying Cai, Wencai Liu, Xintian Cai, Chan Xu, Zhaohui Hu, Xubin Quan, Yizhuo Deng, Hongjie Yao, Binghao Chen, Wenle Li, Chengliang Yin, Qingshan Xu

**Affiliations:** 1 Department of Anesthesiology, Mindong Hospital Affiliated to Fujian Medical University, Fuan, China; 2 Department of Orthopaedic Surgery, the First Affiliated Hospital of Nanchang University, Nanchang, China; 3 Department of Graduate School, Xinjiang Medical University, Urumqi, China; 4 The State Key Laboratory of Molecular Vaccinology and Molecular Diagnostics & Center for Molecular Imaging and Translational Medicine, School of Public Health, Xiamen University, Xiamen, China; 5 Department of Spine Surgery, Liuzhou People’s Hospital, Liuzhou, China; 6 Graduate School of Guangxi Medical University, Nanning, Guangxi China; 7 Guilin Medical University, Guilin, Guangxi China; 8 Faculty of Medicine, Macau University of Science and Technology, Macau, 999078 P. R. China; 9 Department of Orthopaedics, Mindong Hospital Affiliated to Fujian Medical University, Fuan, China

**Keywords:** Osteoporotic vertebral compression fracture, Percutaneous vertebroplasty, Machine learning, Web calculator, Predictive model

## Abstract

**Purpose:**

The aim of this study was to develop and validate a machine learning (ML) model for predicting the risk of new osteoporotic vertebral compression fracture (OVCF) in patients who underwent percutaneous vertebroplasty (PVP) and to create a user-friendly web-based calculator for clinical use.

**Methods:**

A retrospective analysis of patients undergoing percutaneous vertebroplasty: A retrospective analysis of patients treated with PVP between June 2016 and June 2018 at Liuzhou People's Hospital was performed. The independent variables of the model were screened using Boruta and modelled using 9 algorithms. Model performance was assessed using the area under the receiver operating characteristic curve (ROC_AUC), and clinical utility was assessed by clinical decision curve analysis (DCA). The best models were analysed for interpretability using SHapley Additive exPlanations (SHAP) and the models were deployed visually using a web calculator.

**Results:**

Training and test groups were split using time. The SVM model performed best in both the training group tenfold cross-validation (CV) and validation group AUC, with an AUC of 0.77. DCA showed that the model was beneficial to patients in both the training and test sets. A network calculator developed based on the SHAP-based SVM model can be used for clinical risk assessment (https://nicolazhang.shinyapps.io/refracture_shap/).

**Conclusions:**

The SVM-based ML model was effective in predicting the risk of new-onset OVCF after PVP, and the network calculator provides a practical tool for clinical decision-making. This study contributes to personalised care in spinal surgery.

## Introduction

With the economic development of society and medical advances, the life expectancy of people is gradually increasing. And many countries and regions are entering aging societies, and the incidence of osteoporosis has increased significantly [1]. OVCF is an Important complication of age-related osteoporosis [2]. It can cause chronic back pain in the elderly, affecting their mobility and even leading to prolonged bed rest with unpredictable and calamitous consequences [3]. PVP is a minimally invasive surgical procedure that uses bone cement to stabilise the fractured vertebral body and is widely used in the treatment of OVCF due to its advantages in pain relief and restoration of vertebral height [4]. However, PVP is no exception to this rule, and there are two sides to the treatment approach. New OVCF are common in patients with osteoporosis treated with PVP [5]. It either requires reoperation or conservative treatment, which greatly affects the quality life of patients [6].

According to the literature, the incidence of new OVCF ranges from 5.5% to 52.0% [7]. It has also been shown that, OVCF of two or more vertebrae and cement leakage are risk factors for the development of new OVCF after PVP [8, 9]. In addition, some potential risk factors should be further discussed: such as age, gender, BMD, BMI, amount of bone cement injection, and some underlying chronic diseases [3, 9, 10]. The presence of underlying diseases may lead to a longer preoperative preparation time, as well as a longer hospital stay and surgery time. Therefore, it is also informative to include injury to surgery time, hospitalization to surgery time, and surgery time.

ML, a more recent and popular type of artificial intelligence [10], is beginning to be widely used in medical data analysis and in building clinical prediction models [11]. ML algorithms are superior to traditional mathematical and statistical models as predictive tools, and although they may have difficulties with interpretability, they have greater performance in predicting clinical risk. There are no studies using ML algorithms to predict the risk of new-onset OVCF after PVP.T With respect to the application of ML in medicine, the key lies in its clinical utility and interpretability. If these two points are not addressed, there will be significant resistance from clinicians in the application. Currently, common ML visualization applications are the development of web calculators or mobile applications. Yunlang She et al. developed a deep learning model for non-small cell lung cancer survival using the SEER database and an independent validation cohort and visualized it using a user-friendly interface [12]. Liang et al., on the other hand, used deep learning to perform early triage of critically ill patients with COVID-19, incorporated a deep learning model into a nomogram, and used the nomogram to visualize the deep model, providing new ideas for subsequent researchers [13].

In spinal surgery, ML has likewise been partially studied. An intraoperative vascular (IV) injury may be an unavoidable complication of anterior lumbar spine surgery and this intraoperative complication may lead to severe bleeding, thrombosis and postoperative stenosis. Aditya V Karhade et al. developed five ML algorithms for preoperative prediction of VI and trained a natural language processing algorithm for automatic detection of intraoperative VI from [14]. The differential diagnosis of spinal lesions remains challenging even in MRI. a predictive model developed by Vito Chianca et al. using MRI imaging omnipotence combined with ML helped to assess spinal lesions [15]. while Summer S Han et al. developed a ML approach to predict adverse events after spinal surgery [16]. Most referrals for lumbar spine surgery are not for surgery. Identifying surgical candidates early in the referral process can expedite their treatment, while adopting a timely non-operative strategy for those who are unlikely to require surgery. This has important implications for the prognosis of spinal disorders.Nathan Xie et al. constructed an artificial neural network (ANN) to predict surgical candidacy using a model containing eight clinical and imaging predictor variables [17]. Surgery for adult spinal deformities has good outcomes; however, it has high complication and readmission rates.Deeptee Jain et al. developed an integrated model using multivariate logistic regression, random forest, and elastic network regression to predict postoperative discharge, 90-day readmission, and 90-day medical complications. This study can be used to guide surgeon and patient decision making [18]. In recent years, anterior cervical fusion and discectomy (ACDF) performed in an outpatient surgical setting has become popular. Kevin Y Wang et al. developed an ANN that uses preoperative variables to identify patients who may be suitable for outpatient ACDF [19]. Spinal surgery is a high risk event for continued opioid use postoperatively. aditya V Karhade et al. developed ML algorithms for preoperative prediction of the risk of continued opioid prescription after ACDF [20]. Degenerative spinal cord cervical spondylosis is the most common cause of spinal cord dysfunction worldwide. omar Khan et al. developed ML algorithms for predicting the phenotype of patients with mild spinal cord disease who would benefit most from surgery [21].

Therefore, the aim of this study was to develop ML-based models to predict the risk of personalized new-onset OVCF using preoperative and intraoperative clinical features and to build a web calculator.

## Method

### Patients

A retrospective survey of patients admitted to the Department of Spine Surgery of Liuzhou People's Hospital affiliated with Guangxi Medical University from June 2016 to June 2018 who underwent PVP surgery was conducted. This study was approved by the Institutional Review Board of Liuzhou People's Hospital, IRB approval number 2020 (KY-E-22–01)) before data collection and analysis. The Liuzhou People's Hospital Institutional Review Board waived the requirement for informed consent.

### Criteria for new OVCF recognition

This study confirmed the diagnosis by a combination of imaging and signs and symptoms. Recurrence in patients with chest and low back pain was associated with significant pressure pain. x-rays possessed wedge-shaped changes in the corresponding areas of the OVCF, and magnetic resonance imaging (MRI) confirmed the presence of new fractures, where MRI had low signal on T1-weighted images and high signal on T2-weighted images. MRI is also used to rule out diseases with similar symptoms in the spine, including infections and malignancies.

### Data collection

Inclusion criteria included (1) primary osteoporosis with bone density meeting the World Health Organization's diagnostic criteria for osteoporosis; (2) pain or local pressure consistent with imaging findings; and (3) new fractures detected on preoperative spinal X-ray and MRI findings. Exclusion criteria were (1) non-osteoporotic vertebral compression fracture or pathological vertebral compression fracture; (2) treatment with conservative therapy without vertebroplasty.

For each patient, we recorded median values and interquartile ranges (IQR) for age, height, weight, body mass index (BMI), and bone mineral density (BMD), as well as the median injection volume of bone cement used during surgery. Additionally, we noted the presence or absence of cement leakage, the median time from hospitalization to surgery, and the median time from injury to surgery. We also cataloged whether patients had received anti-osteoporosis treatment, had multiple vertebral fractures at baseline, or were undergoing steroid therapy.

### Data baseline and correlation analysis

A baseline was drawn between patients in the no new compression fracture group and those in the new compression fracture group. A chi-square test and t-test were used to compare the differences between these two groups. Heat maps were plotted as correlations for each feature, and heat maps of data distribution were plotted as distributions of features.

### Univariate and multivariate logistic regression analysis

Logistic regression was applied to analyze the relationship between characteristics in 58 new patients with OVCF. We screened for features with a *P* value < 0.05 in univariate logistic regression.

### Training and test set splitting

This study splits the training and test sets based on time. Specifically, the data between June 2016 and June 2017 is used as the training set, while the data between July 2017 and June 2018 is used as the test set. This time-series division helps to better simulate the application of the model in the real world.

### Variable screening

The Boruta algorithm is used for variable selection. This is a fully correlated feature selection method that extends the Random Forest classification method. Unlike random probes, it works by iteratively removing the least important features. We use Boruta to identify the most relevant features from the training set that contribute significantly to the new OVCF prediction. This process ensures that only variables with true predictive power are included in subsequent modeling.

### Prediction models

ML algorithms outperform traditional regression methods in terms of the predictive power of models built from discrete data [22–24]. In this study, we have selected nine different ML algorithms: logistic regression, decision tree (DT), random forest (RF), extreme gradient boosting (XGBoost), light gradient boosting machine (LightGBM), elastic network K-Nearest Neighbors (KNN), Support Vector Machines (SVM), and Multi-Layer Perceptual Machines (MLP). These algorithms were chosen to fully consider the performance of different types of models on the dataset. A tenfold cross validation (CV) approach was used on the training set to ensure the robustness and generalization ability of the models.

### Model performance evaluation and selection

In this study, we first screened the best algorithmic models by ROC_AUC.The AUC serves as an important performance metric that helps us to identify the model that performs the best in distinguishing the risk of novel OVCF. After selecting the best models, we further use DCA to evaluate the performance of the models in terms of clinical utility. DCA is used to ensure that the selected models are not only statistically valid, but also have practical value in actual clinical applications.

### Model visualization and interpretation

After selecting the best model, a web calculator was developed to visualize the predictions of the model. This calculator allows clinicians to enter patient characteristics in order to quickly obtain a prediction of the risk of a novel OVCF. In addition, to improve the transparency and interpretability of the model, we used learning curves and SHAP (SHapley Additive exPlanations) values to interpret the model. The learning curve reveals the performance of the model under different training set sizes, while the SHAP values are used to explain the contribution of each feature to the model's predictions, which helps to understand how the model makes its predictions.

### Statistical analysis and software

Statistical analyses, baseline tables, heat maps, ML models and network calculators were constructed and analyzed using R software (version 4.3.2). p less than 0.05 was considered statistically different.

## Results

### Data baseline and heat map

A total of 385 patients met the inclusion criteria, including 58 patients with new-onset postoperative OVCF, and chi-square and independent samples t-tests were performed. The results are shown in Table 1.

**Table 1:** Baseline table of patients with and without new compression fractures

	Level	Overall (*N* = 385)	No (*N* = 327)	Yes(*N* = 58)	*p*
Age(year) (median [IQR])	NA	75.400 [68.300, 80.700]	74.900 [67.750, 81.100]	76.950 [71.150, 79.275]	0.3942
Sex (%)	Male	77 (20.0)	68 (20.8)	9 (15.5)	0.454
	Female	308 (80.0)	259 (79.2)	49 (84.5)	
High(cm) (median [IQR])	NA	155.000 [149.000, 161.000]	155.000 [148.500, 161.000]	154.500 [150.000, 160.000]	0.7463
weigh (median [IQR])	NA	47.000 [40.000, 58.000]	44.000 [40.000, 57.500]	53.000 [47.000, 62.000]	0.0001
BMI (median [IQR])	NA	19.899 [17.116, 23.873]	19.025 [16.880, 23.237]	23.083 [20.966, 24.604]	< 0.0001
BMD (median [IQR])	NA	4.400 [3.900, 5.000]	4.400 [3.900, 5.000]	4.600 [4.225, 5.175]	0.0063
Injection volume of bone cement(ml) (median [IQR])	NA	4.000 [3.500, 5.000]	4.000 [3.500, 5.000]	4.000 [3.500, 5.000]	0.9773
Leakage (%)	No	304 (78.96)	260 (79.51)	44 (75.86)	0.6502
	Yes	81 (21.04)	67 (20.49)	14 (24.14)	
Hospital stay to surgery(day) (median [IQR])	NA	5.000 [4.000, 6.000]	5.000 [3.000, 6.000]	5.500 [4.000, 6.750]	0.0416
Injury to surgery(day) (median [IQR])	NA	14 [8.000, 30.000]	14.000 [8.000, 30.000]	14.500 [8.000, 34.250]	0.8092
Antiosteoporosis (%)	No	245 (63.64)	199 (60.86)	46 (79.31)	0.0109
	Yes	140 (36.36)	128 (39.14)	12 (20.69)	
Multiple (%)	No	205 (53.25)	185 (56.57)	20 (34.48)	0.003
	Yes	180 (46.75)	142 (43.43)	38 (65.52)	
Steroid (%)	No	320 (83.12)	281 (85.93)	39 (67.24)	0.0009
	Yes	65 (16.88)	46 (14.07)	19 (32.76)	

The mean age of patients in the no new-onset OVCF group was 74.90 years and 76.95 years in the new-onset group, with no statistical difference (*P* = 0.394). In the group without new-onset OVCF, the proportion of male patients was slightly higher than that of patients with new-onset OVCF. For the other patients in the two groups, there were no significant differences in height, cement injection volume, leakage, or surgical injury. There was a significant difference between these two groups in terms of weight and BMI. The new OVCF group had a higher degree of osteoporosis (lower BMD) and a lower rate of use of standard anti-osteoporosis treatment. Also, a higher proportion of the new OVCF group had initial OVCF as multiple vertebral fractures and used steroids (Table 1).

**Fig. 1:**
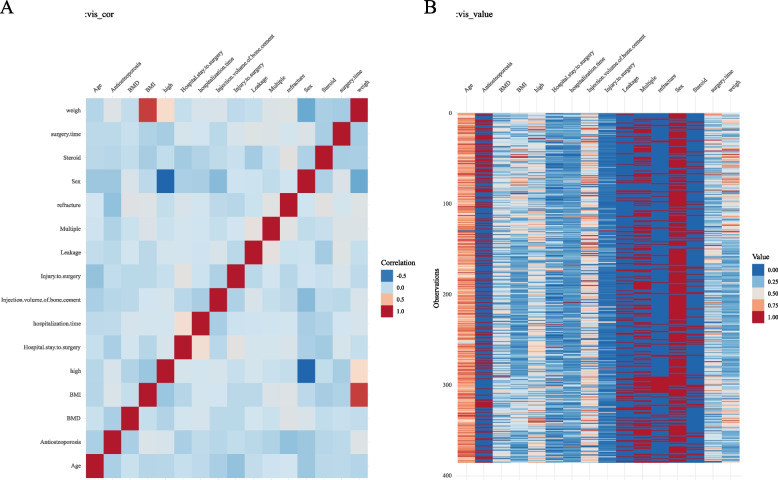
Heat map of correlation and data distribution

The correlation heat map (Fig. 1A) showed that body weight was correlated with BMI and had some correlation with height. The data distribution heat map (Fig. 1B) allows visualization of the distribution of relevant characteristics of the patients.

### Univariate and multivariate logistic regression analyses of new OVCF

The findings of the logistic regression analysis are shown in Table 2. univariate logistic analysis clarified that BMI, BMD, postoperative use of anti-osteoporotic therapy, multiple vertebral fractures, and use of steroid medications were associated with risk factors for new OVCF after surgery (*p* < 0.05). In a multifactorial logistic regression analysis, patients with BMI and osteoporosis were at greater risk. Patients with multiple vertebral fractures in primary OVCF and those using steroid medications were at higher risk. In addition, patients with standardized postoperative use of anti-osteoporosis were at lower risk. Thus, the five predictors of body mass index (BMI), bone mineral density (BMD), multiple vertebral fractures at the time of primary OVCF, lack of anti-osteoporotic therapy, and steroid use are independent risk factors for new-onset OVCF after surgery.

**Table 2:** Univariate and multivariate logistic regression of new vertebral compression fractures

Characteristics	Univariate			Multivariate		
	OR	95%CI	*P*	OR	95%CI	*P*
Age	1.02	0.99–1.05	0.25	NA	NA	NA
Antiosteoporosis1	0.41	0.21–0.79	0.01	0.38	0.19–0.78	0.0083
BMD	1.92	1.24–2.97	< 0.001	1.95	1.22–3.12	0.0051
BMI	1.07	1.02–1.12	0.01	1.09	1.03–1.15	0.0021
Hospital stay to surgery	1.08	1–1.17	0.06	NA	NA	NA
Hospitalization time	1.03	0.98–1.09	0.22	NA	NA	NA
Injection volume of bone cement	0.98	0.75–1.28	0.86	NA	NA	NA
Injury to surgery	1	0.99–1.01	0.63	NA	NA	NA
Surgery time	1.01	1–1.02	0.19	NA	NA	NA
Leakage						
No	Ref	Ref	Ref	Ref	Ref	Ref
Yes	1.23	0.64–2.39	0.53	NA	NA	NA
Multiple						
Only one	Ref	Ref	Ref	Ref	Ref	Ref
More	2.48	1.38–4.44	< 0.001	2.15	1.16–3.99	0.0149
Sex						
Male	Ref	Ref	Ref	Ref	Ref	Ref
Female	1.43	0.67–3.05	0.36	NA	NA	NA
Steroid						
No	Ref	Ref	Ref	Ref	Ref	Ref
Yes	2.98	1.58–5.59	< 0.001	3.58	1.79–7.13	0.0003

### Boruta variable screening

**Fig. 2:**
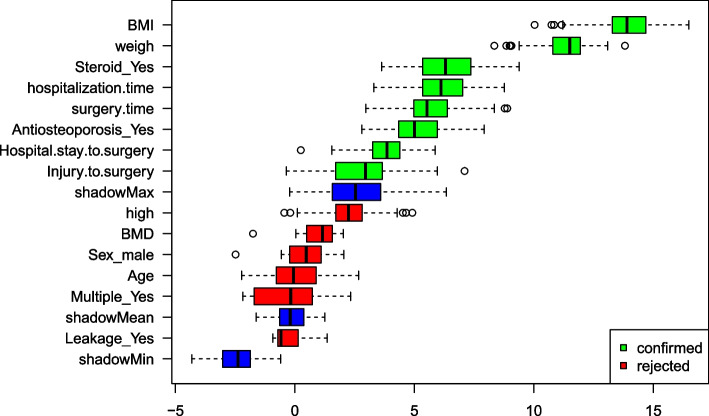
Boruta algorithm for filtering variable results

In our study, feature screening is performed using the Boruta algorithm of the Random Forest framework. This method iteratively compares the importance of each variable with randomly shaded attributes to identify significant predictors. In Fig. 2, green colours are the variables included in the algorithm and red colours are the final excluded variables. In the algorithm was able to effectively differentiate between key predictors such as body mass index, length of hospital stay, duration of surgery and use of anti-osteoporosis medication, ensuring that only variables with strong predictive power were included in the final model. This rigorous screening process underpins the predictive reliability and robustness of our model, highlighting the algorithm's ability to identify the most relevant factors for accurate prediction.

### Model development and validation

**Fig. 3:**
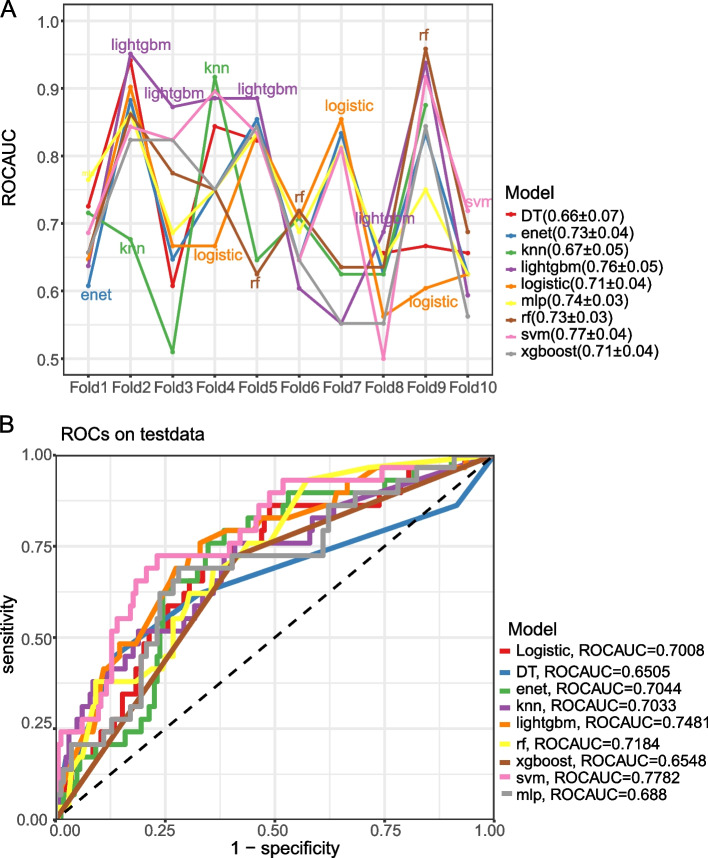
ROC curves of nine ML algorithm models in predicting new OVCF. **A** Machine learning model cross-validation performance in the training set. The line graph represents the area under the receiver operating characteristic curve (ROC AUC) of various machine learning models over ten validations. Each line corresponds to a different model, and the ROC AUC metric indicates the model's ability to distinguish between categories. The legend summarises the mean and standard deviation of the ROC AUC for each model, i.e., Decision Tree (DT), Elastic Net (enet), K-Nearest Neighbours (knn), Light Gradient Boosting Machine (lightgbm), Logistic Regression (logistic), Multi-layer Perceptron (mlp), Random Forest (rf), Support Vector Machine (svm) and Extreme Gradient Boosting ( xgboost). **B** Receiver Operating Characteristic Curves (ROC) for the Test Group This figure shows the ROC curves for each machine learning model, demonstrating their performance on the test dataset.The ROC curves show the true positive rate (sensitivity) and the false positive rate (1—specificity) for different decision thresholds. The diagonal dashed line represents the undifferentiated line. The right-hand legend shows the ROC AUC for each model, illustrating their discriminatory power in predicting new osteoporotic vertebral compression fractures after percutaneous vertebroplasty

After variable screening using boruta, a total of eight independent variables were included and model development was initiated. A comprehensive tenfold CV validation of the nine ML algorithms was performed on the training set as shown in Fig. 3A. This rigorous validation approach revealed the variability and performance consistency of each model, with ensemble techniques such as LightGBM, RF, and SVM exhibiting excellent and consistent AUC scores. In contrast, models such as DT and MLP exhibited greater performance fluctuations across folds, reflecting potential overfitting and sensitivity to training data diversity.

Once in the validation phase, the discriminative power of the models was visually assessed by performing a ROC_AUC analysis on the external test set.The SVM model had the highest AUC, which demonstrated its superior ability to discriminate patient prognosis. This was closely followed by the LightGBM model and the RF model, which also showed high predictive accuracy, but fell short of the accuracy of the SVM. The results are shown in Fig. 3B

Combining the conclusions drawn from the internal tenfold CV and external validation, SVM is considered the best model, which will be further analysed and visualised for interpretation purposes.

### Interpretable analysis of the best model

**Fig. 4:**
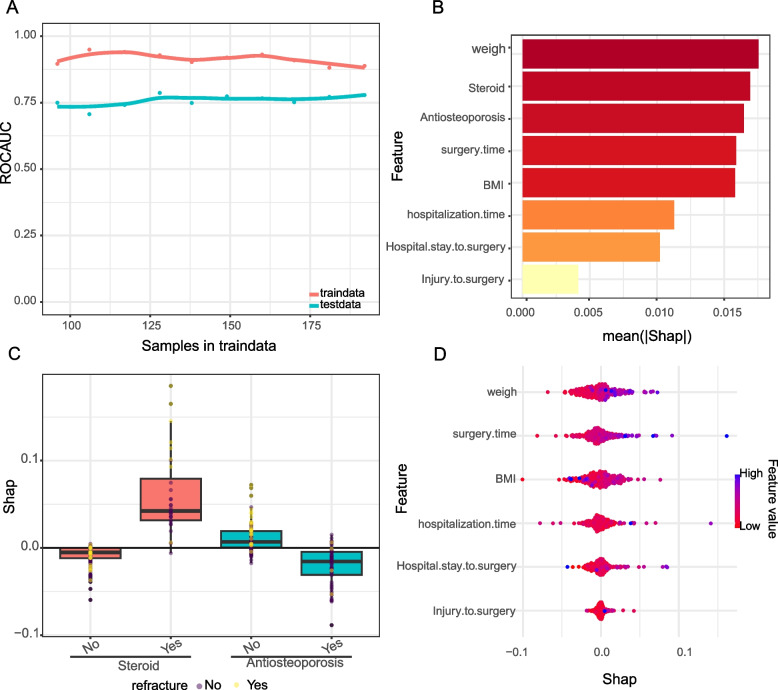
Interpretability of models. **A** Learning curve analysis. This figure depicts the learning curve of the machine learning model, with the Y-axis being ROC_AUC and the X-axis being the number of samples in the training data. The red line represents the performance of the model on the training data and the blue line represents the performance on the test data, indicating how well the model generalises to the unknown data as the number of training samples increases. **B** Feature Importance Bar Graph. The bar chart shows the average SHAP value for each feature, indicating their average impact on the model output. The longer the bar, the greater the impact of the feature on the model prediction. **C** Box plot of SHAP values by fracture feature.The distribution of SHAP values shows the variability of the impact of each feature on the model prediction of a case. **D** Swarm plot of SHAP values. This plot visualises the individual SHAP values for each feature as dots in order to observe the distribution and density of the impact of each feature on the model output. The points are coloured according to the feature value, with pink indicating high values and blue indicating low values, indicating the increasing or decreasing effect of each feature value on the predicted risk of fracture

Figure 4A illustrates the learning curve score of the best model, which depicts the model's ability to learn from the training data, comparing the performance of the training set with that of the independent test set. The curve for the training set shows a high AUC that remains stable after the initial iteration, indicating that the model is proficient at capturing the underlying data distribution. The performance of the test set is slightly lower but remains consistent, indicating that the model generalizes well and does not overfit.

The model's feature importance ranking is demonstrated in Fig. 4B, where the bars rank the features based on their average absolute SHAP value. 'Weight' is the most influential feature, with 'Steroid use' and 'Anti-osteoporosis medication' also having significant effects. This ranking clearly indicates which variables have the greatest impact on model predictions and can be considered as key variables in the decision making process.

Figure 4C and D show the distribution of SHAP values for categorical and continuous variables, respectively. These plots provide an understanding of how the individual values of each feature affect the model output. For example, higher weights and longer operative times positively affect the model's prediction of a higher risk of postoperative complications. Color coding reflects the raw feature values, with red indicating higher values and blue indicating lower values, to allow for an intuitive visual interpretation of the data.

### Clinical applicability

**Fig. 5:**
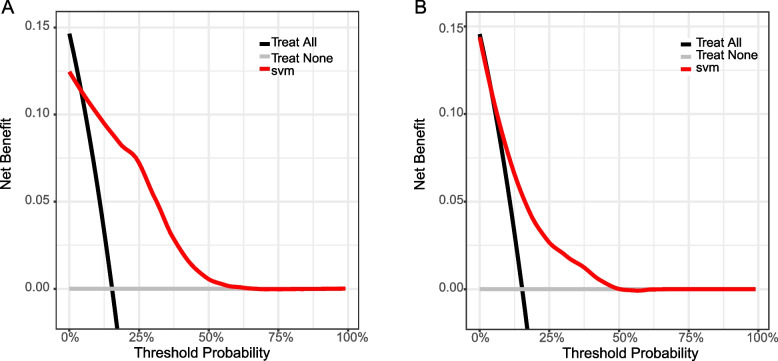
Clinical Decision Curves (DCA) for training and test sets. The decision curve shows the net benefit of the SVM model across different threshold probabilities for the training dataset. The black line represents the net benefit when all patients are treated, and the gray line represents the net benefit when no patients are treated. The red line indicates the net benefit of using the SVM model to decide on treatment, demonstrating its utility over a range of threshold probabilities

Figure 5A and B illustrate the decision curve analysis (DCA) of the SVM model applied to the training and validation datasets, respectively.DCA is a methodology used to evaluate clinical predictive models that calculates the net benefit at different threshold probabilities. These curves compare the net benefit of making a clinical decision using an SVM model with the default strategy of treating all or no patients. In both graphs, the SVM model curves show higher net gains across a range of threshold probabilities compared to the no-treatment and all-treatment strategies, suggesting that the model has clinical value. Notably, the net benefit of the model is most pronounced in the lower threshold probability range, where the decision to intervene is more conservative. This suggests that the SVM model is particularly useful in the clinical setting for identifying patients at risk for postoperative complications so that targeted interventions can be taken early. By quantifying the benefits of the SVM model across a range of potential clinical actions, DCA solidifies the applicability of the SVM model in clinical decision-making.

### Web-based calculator

**Fig. 6:**
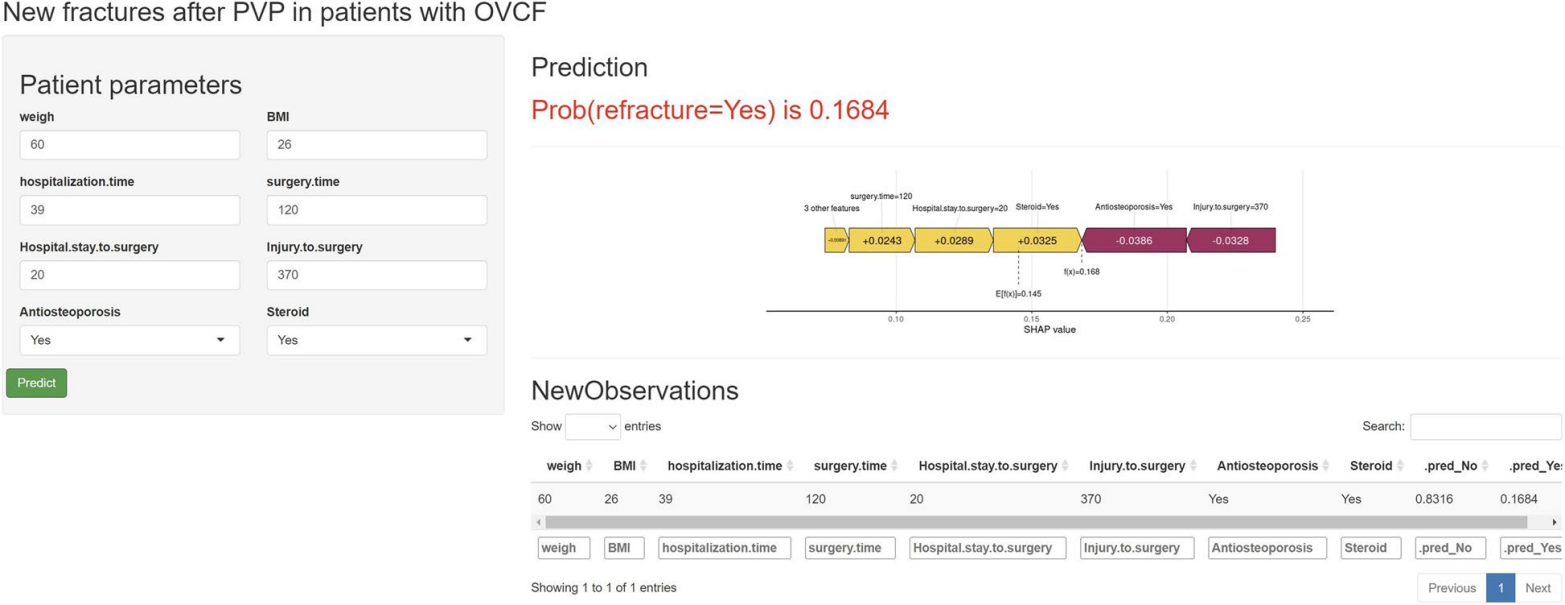
Web calculator interface and SHAP value analysis for OVCF risk prediction. The interface shows input fields for patient parameters relevant to the prediction of new fractures after PVP in patients with OVCF. After entering these parameters and clicking "Predict," the model calculates the probability of a fracture occurring and displays it along with a graph of SHAP values to the right.The SHAP value graph illustrates the contribution of each parameter to the model's prediction, with a positive SHAP value indicating an increased risk of a fracture and a negative SHAP value indicating a decreased risk of a fracture. The length of each bar indicates the level of influence of the feature, and the colour indicates the direction of influence (purple for positive influence, yellow for negative influence)

Figure 6 shows the deployment of the SVM model's web-based calculator, which integrates SHAP)values to assign specific predicted importance values to each feature(https://nicolazhang.shinyapps.io/refracture_shap/). The user-friendly interface prompts the healthcare provider to enter patient-specific data, including weight, body mass index, length of hospitalization, and details of various surgeries, as well as whether treatments such as anti-osteoporosis medications or steroids were used. After the healthcare provider submits new patient-related data, the calculator processes these inputs through a ML model and outputs a probability score. In this case, it indicates a 16.84% chance of a fracture. This predictive probability is a nuanced calculation that takes into account the baseline risk and the individual contributions of each input parameter.The SHAP value plot visually expresses how each feature shifts the probability from the baseline value (in this case 0.168, which represents the average predicted value of the training dataset). For example, longer surgery times and steroid use appear to increase the risk (positive SHAP value), while anti-osteoporosis treatment reduces the risk of new fractures (negative SHAP value).

## Discussion

In this study, we developed and validated several commonly used ML algorithms for predicting the risk of new compression fractures in OVCF patients after PVP. A comparison of the ML algorithms revealed that the SVM model had the best performance. To make the model more user-friendly, we further developed a web-based calculator for interpreting ML predictions using SHAP values for estimating the individual probability of a new compression fracture in OVCF patients treated with PVP. The calculator aids in medical and intraoperative decision-making and is valuable in postoperative follow-up and prevention. Spine surgeons can quickly calculate the risk of a new compression fracture by inputting patient-specific parameters such as weight, body mass index, and surgical details. This is more than just a probabilistic output; the SHAP value clearly explains the impact of each factor on risk. This detailed insight enables clinicians to prioritize which risk factors to address and how to modify treatment strategies. Specifically, adjustments to the dose of anti-osteoporosis medications can be considered, along with other therapeutic interventions to mitigate risk.

Moreover, the inclusion of the Boruta algorithm, a feature selection method, enhances our model by identifying the most significant features impacting the risk of new-onset OVCF. This algorithm works by comparing the importance of real features with that of shadow features, which are generated by shuffling the values of the real features. In our analysis, highlighted in Fig. 2, green boxes represent the important features identified by Boruta, such as BMI, weight, steroid use, length of hospital stay, and surgery duration, while red boxes indicate features deemed unimportant. This method consistently showed that key factors like BMI and weight had a strong influence on model predictions, surpassing their shadow counterparts in importance.

Combining the insights from Boruta analysis with logistic regression results and SHAP analysis provides a comprehensive view of the factors influencing the risk of new-onset OVCF. The Boruta algorithm's identification of critical factors reinforces their significant role in the model's predictions. Conversely, features represented at the bottom of the graph, such as shadowMin (potentially the lowest value of a feature in the entire dataset), were found to be less impactful. This multifaceted approach, integrating Boruta analysis with other predictive tools, offers a robust framework for understanding and mitigating the risk of new compression fractures in OVCF patients.

From a logistic regression perspective, weight and BMI may exhibit strong covariance, as indicated by the correlation heatmap, necessitating their removal to avoid analysis interference [25]. This action is supported by findings that identify BMI and steroid use as statistically significant predictors of new-onset OVCF, aligning with Boruta's analysis which also highlights steroid use as a significant factor. However, logistic regression contrasts with Boruta by not considering the length of hospital stay and surgery duration as significant predictors. The utilization of SHAP values further enriches our understanding by quantifying the influence of each characteristic on individual predictions, for instance, demonstrating how a high BMI may elevate the risk of new-onset OVCF. This comprehensive approach, integrating logistic regression with Boruta and SHAP analyses, enables clinicians to prioritize interventions with greater precision, particularly for conditions like high BMI and steroid use, which are confirmed as critical factors across analyses [26].

Unlike traditional logistic regression that offers a broad risk overview based on population averages, our study introduces an enhanced ML model incorporating SHAP values for a more nuanced risk assessment. This model is adept at revealing critical interactions, such as those between age and other factors like BMD or prior steroid use, which might not be evident through conventional statistical methods. Such detailed insights allow for the development of targeted interventions, acknowledging the complex, nonlinear patterns and high-dimensional interactions characteristic of healthcare data.

Fracture is fundamentally a mechanical failure, and bone density, bone mass, and mechanical properties of the vertebral body play key roles in fracture risk assessment. Incorporating biomechanical modeling into our predictive framework offers a more comprehensive view of fracture risk, thus enhancing the accuracy and applicability of our predictions. Biomechanical models provide detailed insights into skeletal load-bearing capacity and structural integrity, enabling more precise determinations of fracture probability under varying biomechanical conditions. This approach is particularly relevant for assessing the risk of vertebral compression fractures, where factors such as BMD, bone quality assessments, and microstructural damage significantly influence fracture susceptibility [27]. Furthermore, the consideration of differences in bone quality and mechanical characteristics among patients allows for the individualized tailoring of treatment strategies. For instance, patients with lower bone density or poorer bone quality may require more aggressive anti-osteoporotic therapies and special considerations during procedures such as vertebroplasty [28]. Modeling the loading conditions and stress distribution within the vertebral body enables the prediction of potential fracture locations and identification of vulnerable areas under various biomechanical scenarios. This not only aids in understanding fracture mechanisms but also guides surgical implant placement and postoperative rehabilitation planning to minimize subsequent fracture risks [29]. Moreover, analyzing how changes in bone properties specifically affect fracture risk deepens our understanding of how factors such as age, gender, lifestyle, and genetics indirectly influence fracture susceptibility by influencing bone mechanical properties. This forms the basis for developing comprehensive prevention and treatment strategies, particularly for high-risk populations [30]. In summary, integrating mechanical factors and biomechanical models into our predictive framework provides a more comprehensive and in-depth perspective on fracture risk, facilitating the development of precise and personalized prevention and treatment plans. This interdisciplinary approach, merging machine learning techniques with biomechanical research, offers a scientific and comprehensive risk assessment tool for OVCF patients. Building on these analytical advancements, our research presents a novel contribution: a web-based calculator designed to assess the risk of new-onset OVCF using clinical data, explained through SHAP values [31, 32]. This tool offers a significant leap forward in postoperative prevention and treatment planning, enabling clinicians to formulate individualized treatment strategies. Accessible via web-connected devices, this calculator represents a more accurate and cost-effective solution than traditional scoring methods, facilitating real-time, data-driven decision-making during clinical care.

However, there are limitations to this study. First, retrospective studies may lead to selection bias. Second, the ML algorithm model we built was single-agency, and although we attempted to use time as a division between training and testing groups, this may limit its generalisability and should be further validated in real-world data. Also, fracture is a mechanical event, and we should also consider adding some relevant studies and risk expansion models in the future. In the future, we should conduct multicentre studies to expand data sources and keep updating the model.

## Conclusions

Our study has developed and validated an ML algorithm for predicting new-onset OVCF in OVCF patients treated with PVP using readily available relevant variables. The predictive model based on the ML algorithm can accurately identify whether a patient is at high risk. The web calculator can be used as an available tool for clinicians to make accurate medical decisions, and in the future we aim to integrate more data and evidence, such as image data, through multiple centers to improve.

## Data Availability

The original contributions presented in the study are included in the article, further inquiries can be directed to the corresponding author/s.

## References

[1] Coughlan T, Dockery F. Osteoporosis and fracture risk in older people. Clin Med (Lond). 2014;14(2):187–91. 10.7861/clinmedicine.14-2-187. 10.7861/clinmedicine.14-2-187 PMC4953292 24715132

[2] Zhang H, Xu C, Zhang T, Gao Z, Zhang T (2017). Does Percutaneous Vertebroplasty or Balloon Kyphoplasty for Osteoporotic Vertebral Compression Fractures Increase the Incidence of New Vertebral Fractures? A Meta-Analysis. Pain Physician.

[3] Chen Z, Chen Z, Wu Y, Wu Y, Ning S, Ning S, Ma T, Ma T, Wu Z, Wu Z. Risk Factors of Secondary Vertebral Compression Fracture After Percutaneous Vertebroplasty or Kyphoplasty: A Retrospective Study of 650 Patients. Med Sci Monit. 2019;25:9255–61. 10.12659/MSM.915312. 10.12659/MSM.915312 PMC6911304 31740653

[4] Filippiadis DK, Marcia S, Masala S, Deschamps F, Kelekis A. Percutaneous Vertebroplasty and Kyphoplasty: Current Status, New Developments and Old Controversies. Cardiovasc Intervent Radiol. 2017;40(12):1815–23. 10.1007/s00270-017-1779-x. 10.1007/s00270-017-1779-x 28856402

[5] Lohle P, Juttmann JR, Voormolen M, Yolanda V, Fransen H, Lampmann L (2006). The risk of new osteoporotic vertebral compression fractures in the year after percutaneous vertebroplasty. J Vasc Interv Radiol.

[6] Klazen C, Venmans A, Vries JD, Rooij W, Verhaar HJJ (2010). Percutaneous vertebroplasty is not a risk factor for new osteoporotic compression fractures: results from VERTOS II. Ajnr Am J Neuroradiol.

[7] Lindsay R, Burge RT, Strauss DM (2005). One year outcomes and costs following a vertebral fracture. Osteoporos Int.

[8] Li H, Yang DL, Ma L, Wang H, Yang SD (2017). Risk Factors Associated with Adjacent Vertebral Compression Fracture Following Percutaneous Vertebroplasty After Menopause: A Retrospective Study. Med Sci Monit.

[9] Lin D, Hao J, Lin L, Lei W, Lian K (2017). Effect of Bone Cement Volume Fraction on Adjacent Vertebral Fractures After Unilateral Percutaneous Kyphoplasty. Clin Spine Surg.

[10] Tseng YY, Yang TC, Tu PH, Lo YL, Yang ST. Repeated and multiple new vertebral compression fractures after percutaneous transpedicular vertebroplasty. Spine (Phila Pa 1976). 2009;34(18):1917–22. 10.1097/BRS.0b013e3181ac8f07. 10.1097/BRS.0b013e3181ac8f07 19652633

[11] Wu EQ, Hu D,  Deng PY, Tang Z, Ren H (2020). Nonparametric Bayesian Prior Inducing Deep Network for Automatic Detection of Cognitive Status. IEEE Trans Cybern.

[12] She Y, Jin Z, Wu J, Deng J, Zhang L, Su H, Jiang G, Liu H, Xie D, Cao N (2020). Development and Validation of a Deep Learning Model for Non-Small Cell Lung Cancer Survival. JAMA Netw Open.

[13] Liang W, Yao J, Chen A, Lv Q, Zanin M, Liu J, Wong S, Li Y, Lu J, Liang H (2020). Early triage of critically ill COVID-19 patients using deep learning. Nat Commun.

[14] Karhade AV, Bongers MER, Groot OQ, Cha TD, Doorly TP, Fogel HA, Hershman SH, Tobert DG, Srivastava SD, Bono CM (2021). Development of machine learning and natural language processing algorithms for preoperative prediction and automated identification of intraoperative vascular injury in anterior lumbar spine surgery. Spine J.

[15] Chianca V, Cuocolo R, Gitto S, Albano D, Merli I, Badalyan J, Cortese MC, Messina C, Luzzati A, Parafioriti A (2021). Radiomic Machine Learning Classifiers in Spine Bone Tumors: A Multi-Software. Eur J Radiol.

[16] Han SS, Azad TD, Suarez PA, Ratliff JK (2019). A machine learning approach for predictive models of adverse events following spine surgery. Spine J.

[17] Xie N, Wilson PJ, Reddy R. Use of machine learning to model surgical decision-making in lumbar spine surgery. Eur Spine J. 2022;31(8):2000–6. 10.1007/s00586-021-07104-8. 10.1007/s00586-021-07104-8 35088119

[18] Jain D, Durand W, Burch S, Daniels A, Berven S (2020). Machine Learning for Predictive Modeling of 90-day Readmission, Major Medical Complication, and Discharge to a Facility in Patients Undergoing Long Segment Posterior Lumbar Spine Fusion. Spine.

[19] Wang KY, Suresh KV, Puvanesarajah V, Raad M, Margalit A, Jain A (2021). Using Predictive Modeling and Machine Learning to Identify Patients Appropriate for Outpatient Anterior Cervical Fusion and Discectomy. Spine.

[20] Karhade AV, Ogink PT, Thio Q, Broekman MLD, Cha TD, Hershman SH, Mao J, Peul WC, Schoenfeld AJ, Bono CM (2019). Machine learning for prediction of sustained opioid prescription after anterior cervical discectomy and fusion. Spine J.

[21] Khan O, Badhiwala JH, Witiw CD, Wilson JR, Fehlings MG (2021). Machine learning algorithms for prediction of health-related quality-of-life after surgery for mild degenerative cervical myelopathy. Spine J.

[22] Cai J, Luo J, Wang S, Yang S (2018). Feature selection in machine learning: A new perspective. Neurocomputing.

[23] Ngiam KY, Khor IW (2019). Big data and machine learning algorithms for health-care delivery. Lancet Oncol.

[24] Li W, Dong S, Wang H, Wu R, Wu H, Tang ZR, Zhang J, Hu Z, Yin C (2021). Risk analysis of pulmonary metastasis of chondrosarcoma by establishing and validating a new clinical prediction model: a clinical study based on SEER database. BMC Musculoskelet Disord.

[25] LaValley MP (2008). Logistic regression. Circulation.

[26] Yue S, Li S, Huang X, Liu J, Hou X, Zhao Y, Niu D, Wang Y, Tan W, Wu J (2022). Machine learning for the prediction of acute kidney injury in patients with sepsis. J Transl Med.

[27] Faulkner KG, Cummings SR, Black D, Palermo L, Glüer CC, Genant HK (1993). Simple measurement of femoral geometry predicts hip fracture: the study of osteoporotic fractures. J Bone Miner Res.

[28] Oakland RJ, Furtado NR, Wilcox RK, Timothy J, Hall RM (2009). Preliminary biomechanical evaluation of prophylactic vertebral reinforcement adjacent to vertebroplasty under cyclic loading. Spine J.

[29] Yang C, Wang F, Huang X, Zhang H, Zhang M, Gao J, Shi S, Wang F, Yang F, Yu X (2023). Finite element analysis of biomechanical effects of mineralized collagen modified bone cement on adjacent vertebral body after vertebroplasty. Front Bioeng Biotechnol.

[30] Seeman E, Delmas PD (2006). Bone quality–the material and structural basis of bone strength and fragility. N Engl J Med.

[31] Ma Y, Lu Q, Yuan F, Chen H (2023). Comparison of the effectiveness of different machine learning algorithms in predicting new fractures after PKP for osteoporotic vertebral compression fractures. J Orthop Surg Res.

[32] Hu X, Zhu Y, Qian Y, Huang R, Yin S, Zeng Z, ..., Cheng L. Prediction of subsequent osteoporotic vertebral compression fracture on CT radiography via deep learning. View. 2022;3(6):20220012.

